# Risk factors for adverse events associated with trimethoprim-sulfamethoxazole treatment for Pneumocystis pneumonia in non-human immunodeficiency virus-infected patients: a multicenter, retrospective, observational cohort study

**DOI:** 10.1186/s12879-026-12600-7

**Published:** 2026-01-20

**Authors:** Reina Idemitsu, Tatsuya Nagai, Hiroki Matsui, Haruka Fujioka, Yuya Homma, Ayumu Otsuki, Hiroyuki Ito, Shinichiro Ohmura, Toshiaki Miyamoto, Daisuke Shichi, Tomohisa Watari, Yoshihito Otsuka, Kei Nakashima

**Affiliations:** 1 Department of Pulmonology, Kameda Medical Center, 929 Higashi-Cho, Kamogawa, Chiba 296-8602 Japan; 2 Department of Clinical Epidemiology and Health Economics, School of Public Health, the University of Tokyo, Tokyo, Japan; 3 Clinical Research Support Office, Kameda Medical Center, Kamogawa, Chiba Japan; 4 Department of Rheumatology, Seirei Hamamatsu General Hospital, Hamamatsu, Shizuoka Japan; 5 Department of Infectious Diseases and Rheumatology, Seirei Mikatahara General Hospital, Hamamatsu, Shizuoka Japan; 6 Department of Clinical Laboratory, Kameda Medical Center, Kamogawa, Chiba Japan

**Keywords:** Trimethoprim-sulfamethoxazole, Non-human immunodeficiency virus-infected patients, Pneumocystis pneumonia, Adverse events

## Abstract

**Background:**

*Pneumocystis jirovecii* pneumonia (PCP) is a severe opportunistic infection. Trimethoprim-sulfamethoxazole (SXT) is the first-choice treatment for PCP in human immunodeficiency virus (HIV)-infected and non-HIV-infected patients. However, the high incidence of adverse events makes treatment with SXT difficult. The risk factors for these adverse events in patients with non-HIV PCP remain unclear.

**Methods:**

In this multicenter, retrospective, observational cohort study, we investigated these risk factors by analyzing data from patients with non-HIV PCP treated with SXT between June 2006 and March 2021 across three institutions. Patients were divided into two groups based on the presence of grade 3 or higher adverse events according to Common Terminology Criteria for Adverse Events (CTCAE) version 5.0: the adverse event (*n* = 74) and no adverse event (*n* = 62) groups. Patient characteristics were compared, and multivariate regression analysis was used to identify factors contributing to adverse events. We also investigated the relationship between treatment failure and adverse events.

**Results:**

Baseline characteristics showed notable variations between the groups, notably in serum sodium, serum potassium levels, and the initial trimethoprim dose per weight. Logistic regression analysis revealed significant associations among adverse events and these three baseline variables. In the treatment failure group, the most frequent adverse events included skin rashes, hyponatremia, and hyperkalemia.

**Conclusions:**

Pretreatment serum sodium and potassium levels and the initial SXT dose per weight were identified as independent risk factors for developing CTCAE grade 3 or higher adverse events associated with SXT treatment for non-HIV PCP. Further large-scale, prospective studies are essential to confirm these results.

**Supplementary Information:**

The online version contains supplementary material available at 10.1186/s12879-026-12600-7.

## Background

*Pneumocystis jirovecii pneumonia* (PCP) is a significant opportunistic infection predominantly affecting immunocompromised individuals, such as those with AIDS. This severe respiratory disease is associated with a high mortality rate, particularly in untreated cases [1]. Advances in early HIV treatment have substantially decreased the incidence of PCP in this population. The incidence of PCP in non-HIV-infected patients, however, has been increasing owing to the use of biologics, molecular-targeted drugs, anticancer drugs, and increasing rates of organ transplantation [2]. The mortality rate is high in HIV-infected patients with PCP (range, 10–20%). On the other hand, PCP in non-HIV-infected patients progresses rapidly, causing severe respiratory failure with a poor prognosis. The mortality rates among patients with non-HIV PCP are higher than those among those with HIV PCP (range, 30–60%) [1]. Therefore, improving the management of PCP in non-HIV-infected patients is essential to improve prognosis.

SXT remains the primary treatment for both HIV PCP and non-HIV PCP, with recommended doses of trimethoprim ranging from 15 to 20 mg/kg/day and sulfamethoxazole from 75 to 100 mg/kg/day [3, 4]. Despite its efficacy, adverse events such as hematologic toxicity, gastrointestinal disorders, and renal impairment often limit its use [5–7], and switching to alternative therapy is often necessary [8]. Several studies have evaluated the risk of adverse events in patients with HIV PCP treated with SXT and have shown that an increased dose (> 16 mg/kg/day), age [6], duration of treatment, and CD4/8 ratio [9] are risk factors for adverse events. However, the risk factors for adverse events associated with SXT treatment in patients with non-HIV PCP have not been adequately studied. The backgrounds of patients with non-HIV PCP and HIV PCP are very different. Patients with non-HIV PCP are usually older, more often female, and with multiple underlying medical conditions, compared with patients with HIV PCP [10, 11]. Therefore, evaluating the risk factors for adverse events associated with SXT treatment in patients with non-HIV PCP is essential. In this multicenter, retrospective, observational cohort study, we aimed to identify risk factors for adverse events associated with SXT treatment in patients with non-HIV PCP.

## Materials and methods

### Study design and study population

This study was a multicenter, retrospective, observational cohort study targeting non-HIV-infected patients who were clinically diagnosed with PCP and treated with SXT. Non-HIV-infected patients diagnosed with PCP between January 2006 and March 2021 at Kameda General Hospital, Seirei Hamamatsu Hospital, and Seirei Mikatahara Hospital were retrospectively enrolled (the registry from which the data were obtained was RE-VISION-PCP: Registry to Provide New Evidence and Insights for the Management of Pneumocystis Pneumonia in non-HIV-infected Patients) [12]. The three diagnostic criteria for PCP were defined according to diagnostic guidelines for non-HIV PCP and prior research [5, 12–14]; (1) host factor, including potential immunocompromised status besides HIV; (2) clinical criteria comprising signs and symptoms consistent with PCP (such as dyspnea, cough, fever, and hypoxemia), and chest X-ray or computed tomography findings indicative of PCP, such as bilateral or diffuse ground-glass shadows; (3) microbial criteria involving detection of *Pneumocystis jirovecii* in respiratory specimens via conventional staining methods (Grocott methenamine silver or Diff-Quick staining) or deoxyribonucleic acid testing (loop-mediated isothermal amplification or polymerase chain reaction). Additionally, patients with elevated serum β-D-glucan levels combined with a positive response to conventional PCP therapy were included. Serum β-D-glucan level was assessed using either the β-D-glucan test kit (Wako Pure Chemical Industries, Osaka, Japan) or the FUNGITEC G test MKII (Nissui Pharmaceutical, Tokyo, Japan). An elevated β-D-glucan level was defined as > 5 pg/mL (β-D-glucan test; Wako assay) or > 20 pg/mL (FUNGITEC G-test KM assay) [15, 16]. Patients were excluded according to the following criteria: (1) no history of any treatment, (2) initial treatment with regimens other than SXT, and (3) history of receiving > 20 mg/kg of trimethoprim. The study protocol was reviewed and approved by the research ethics committees of Kameda General Hospital (#21-069-230801), Seirei Hamamatsu Hospital (#3584), and Seirei Mikatahara Hospital (#21–58). Patient consent was obtained via an opt-out procedure according to Japanese ethical guidelines, owing to the retrospective nature of this study.

### Standard treatment setting

According to previous guidelines and reviews, the standard dose of SXT is 15–20 mg/kg [3, 4]. The dose administered during the study period was determined by physicians. The standard treatment period was 2–3 weeks, as recommended by the guidelines, and the treatment was continued until the patient’s general and respiratory statuses stabilized [3, 4]. If adverse events made continued treatment difficult, the SXT dose was reduced, or the SXT regimen was changed to intravenous pentamidine (4 mg/kg/day) or oral atovaquone (1,500 mg/day), as recommended by international guidelines [1, 3].

### Definitions of the adverse events of trimethoprim-sulfamethoxazole treatment

The patients were divided into two groups: those with and without adverse events. We compared the patient characteristics between these groups and investigated the factors contributing to the occurrence of adverse events using multivariate regression analysis. Adverse events were categorized as grade 3 or higher according to the Common Terminology Criteria for Adverse Events (CTCAE) version 5.0 [17]. This categorization includes: maculo-papular rash (macules/papules covering > 30% body surface area with moderate or severe symptoms, limiting self-care activities of daily living); fever (> 40.0 °C or > 104.0 °F for ≤ 24 h); anorexia (associated with significant weight loss or malnutrition, e.g., inadequate oral caloric and/or fluid intake, with tube feeding or parenteral nutrition indicated); nausea (requiring tube feeding, parenteral nutrition, or hospitalization due to inadequate oral intake); decreased white blood cell count (< 1,000–2,000/mm^3^ or < 1.0–2.0 × 10^9^/L); anemia (hemoglobin < 8.0 g/dL, < 4.9 mmol/L, or < 80 g/L, with transfusion indicated); decreased platelet count (< 25,000–50,000/mm^3^ or < 25.0–50.0 × 10^9^/L); increased aspartate aminotransferase (AST) and alanine aminotransferase (ALT) (> 5.0–20.0 U/L × upper limit of normal if baseline value was normal; > 5.0–20.0 U/L × baseline value if it was abnormal); increased blood bilirubin (> 3.0–10.0 U/L × upper limit of normal if baseline value was normal; > 3.0–10.0 × baseline value if abnormal); hyponatremia (serum sodium [Na] level 125–129 mmol/L if symptomatic; 120–124 mmol/L regardless of symptoms); hyperkalemia (serum potassium [K] level > 6.0–7.0 mmol/L, with hospitalization indicated).

### Data collection

Following data were obtained retrospectively from the patients’ medical records: age; sex; height; weight; presence of underlying diseases, including malignancy, autoimmune and collagen diseases; laboratory test results at hospital admission (platelet count; serum levels of hemoglobin, albumin, lactate dehydrogenase, sodium, potassium, and creatinine; creatinine clearance [CrCl]); eGFR; types of immunosuppressive drugs used before the occurrence of PCP; types of primary prophylaxis undertaken for PCP; period from onset of symptoms of PCP to the diagnosis of PCP; blood pressure and oxygen dose at the beginning of treatment; blood laboratory data at the beginning of treatment starting dose and duration of treatment with SXT; any treatment interruption due to adverse events; any change from SXT treatment to second-line treatment; any history of corticosteroid treatment; patient characteristics; treatment course; any adverse events; patient outcomes; any history of concomitant corticosteroid treatment, high flow nasal cannula ventilation, noninvasive positive pressure ventilation, and ventilatory management. The dose of SXT was calculated by multiplying the TMP dose per body weight by a correction factor based on CrCl, because dose adjustment or dose reduction was necessary depending on the degree of renal dysfunction. CrCl was calculated using the Cockcroft-Gault formula [18]. Correction coefficients were 1 if CrCl was > 50 mL/min, 1.5 if CrCl was 30–50 mL/min, 2.0 if CrCl was 15–30 mL/min, and 3.0 if CrCl was < 15 mL/min or if the patient was on hemodialysis [19]. We additionally calculated the eGFR using the standard formula recommended by the Japanese Society of Nephrology, which is widely used in Japan [20]. Because SXT dosing and clinical pharmacokinetic labeling are commonly based on CrCl (Cockcroft-Gault formula), we prespecified CrCl as the renal function parameter for the primary analyses; eGFR was calculated post hoc for descriptive purposes. We did not collect data on which specific adverse event was the direct cause of the discontinuation or change.

### Outcomes

The primary outcome was the occurrence of grade 3 or higher adverse events according to CTCAE version 5.0.

### Stratification

We divided the patients into two groups to investigate factors associated with the occurrence of adverse events: the adverse event group and no adverse event group. The adverse events group comprised patients who experienced grade 3 or higher adverse events according to CTCAE version 5.0. The no adverse events group included those who did not experience such adverse events. Additionally, for a secondary analysis to evaluate the relationship between treatment failure and adverse events, we divided the patients into those with and no treatment failure. Treatment failure was defined as discontinuation of the initial SXT therapy or switching to a different agent owing to adverse events. We did not collect data on which specific adverse event was the direct cause of the discontinuation or change.

### Statistical analysis

Because this was a retrospective observational study, it was conducted using the number of available cases, and no pre-study sample size calculation was performed. We divided the eligible patients into adverse event and no adverse event groups and calculated descriptive statistics for baseline characteristics and outcomes. To assess the distribution of continuous variables, we used the Shapiro–Wilk test. Normally distributed variables were summarized as mean ± standard deviation and compared between groups using the t-test, whereas skewed variables were summarized as median [P27–P75] and compared using the Mann–Whitney U test. Categorical variables were compared using Fisher’s exact test. To allow for a more detailed analysis, age was treated as a continuous variable and was also categorized into two groups: <65 years and ≥ 65 years. This classification is based on the common use of 65 years as a standard for demarcating the older population [21]. Logistic regression analysis was used to calculate odds ratios (ORs) and their 95% confidence intervals (CIs) to assess the risk factors for the occurrence of grade 3 or higher adverse events. First, we performed univariable logistic regression analyses for each candidate variable; these results are presented in Supplementary Table 2. For the multivariable model shown in Table 2, we selected seven covariates, prioritizing variables supported by the literature and clinically relevant, with reference to the univariable associations. Specifically, patient background information (e.g., age [6]) and risk factors (serum sodium and potassium levels, renal function parameters, the details of initial treatment [6, 22–24], comorbidities [12] [e.g., malignancy], presence or absence of steroid treatment [6], etc.) determined based on prior research and clinical experience were entered into the logistic regression model. Additionally, to confirm that our findings about the primary risk factors were not dependent on the handling of age, we also conducted a sensitivity analysis that treated age as a categorical variable. Furthermore, as a sensitivity analysis, we applied a data-driven variable selection procedure using the bestglm package with the Akaike information criterion (AIC) in R to construct an alternative predictive model; the results of this model are presented in the Supplementary material. Multicollinearity was assessed using variance inflation factors (VIFs). Model discrimination was evaluated by the c-statistic (area under the ROC curve, AUC), and calibration was assessed using the Hosmer–Lemeshow goodness-of-fit test. The significance level was set at *P* < 0.05. Statistical analyses were performed using R (version 4.3.0; R Development Core Team).

## Results

**Fig. 1:**
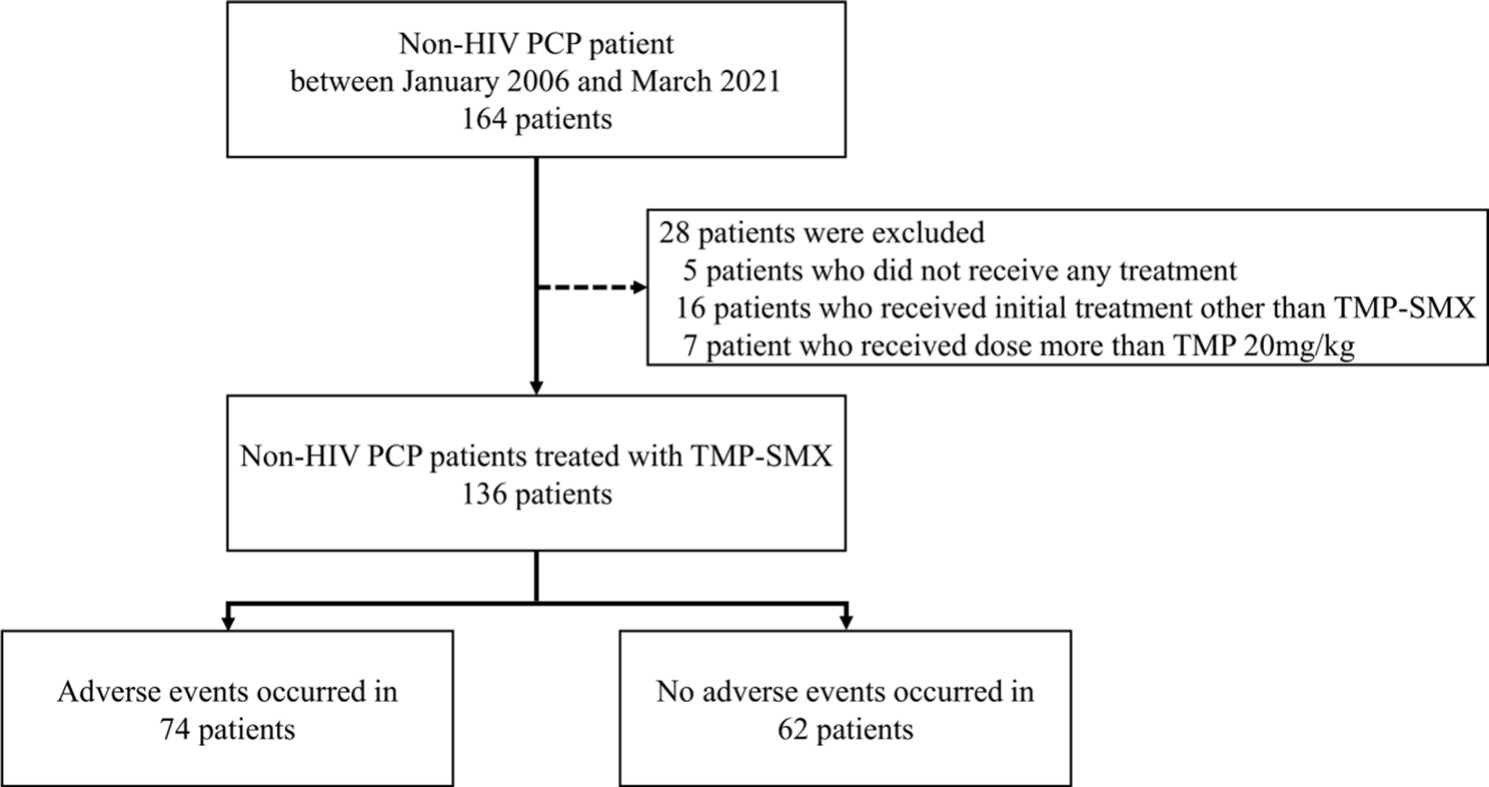
Patient selection flowchart PCP, *Pneumocystis jirovecii* pneumonia; HIV, human immunodeficiency virus; SXT, trimethoprim-sulfamethoxazole

Figure 1 shows the patient selection flowchart. A total of 164 patients diagnosed with non-HIV PCP were included. Five patients who did not receive any treatment, 16 patients who received an initial treatment other than SXT, and seven patients who received a TMP dose of ≥ 20 mg/kg/day were excluded. Finally, 74 patients were included in the adverse event group, and 62 patients were included in the no adverse event group.

Table 1 summarizes the baseline characteristics of adverse events and no adverse event groups according to CTCAE version 5.0. No notable variations were observed in age, sex, or underlying medical diseases. Serum sodium, potassium, and AST levels showed marked differences, while other parameters remained consistent across groups. No notable variations in hypotension (systolic blood pressure < 90 mmHg), respiratory status, or steroid administration were observed. Notable variations were observed in the initial dose of trimethoprim per weight. Details of baseline immunosuppressants and biologic immunosuppressive medications and adjunctive corticosteroid use during PCP treatment, stratified by adverse events, are shown in Supplementary Table 1.

**Table 1:** Baseline clinical characteristics of patients with and without adverse events associated with trimethoprim-sulfamethoxazole treatment

Variable	No adverse events (*n* = 74)	Adverse events (*n* = 62)	*P* value
Age (years)	71.50 [64.50, 78.75]	72.5 [63.00, 77.75]	0.760
Age Category, n (%)			0.847
Age < 65 years	19 (25.7)	17 (27.4)	
Age ≥ 65 years	55 (74.3)	45 (72.6)	
Women, n (%)	40 (54.1)	35 (56.5)	0.863
Weight (kg)	53.70 (± 11.35)	51.27 (± 11.06)	0.211
Underlying diseases			
Malignancy, n (%)	20 (27.0)	10 (16.1)	0.149
Hematologic malignancy, n (%)	10 (13.5)	3 (4.8)	0.141
Solid tumor, n (%)	11 (14.9)	7 (11.3)	0.617
Connective tissue disease, n (%)	51 (68.9)	48 (77.4)	0.334
Prophylaxis for PCP	2 (2.7)	2 (3.2)	> 0.99
SXT	2 (2.7)	0 (0.0)	
Pentamidine	0 (0.0)	2 (3.2)	
Blood test findings			
White blood cell count (/µL)	8,060.00 [5922.50, 9912.50]	8250.00 [5242.50, 10815.00]	0.955
Lymphocyte (%)	10.10 [6.50, 16.00]	13.20 [7.32, 20.50]	0.129
Hemoglobin (g/dL)	11.57 (± 1.83)	11.45 (± 2.28)	0.734
Platelet count (× 10⁴/µL)	21.55 [15.00, 25.78]	21.75 [15.90, 30.30]	0.256
Albumin (g/dL)	2.89 (± 0.63)	3.04 (± 0.69)	0.205
Lactate dehydrogenase (IU/L)	347.50 [288.75, 454.75]	383.50 [292.25, 474.00]	0.467
Sodium (mEq/L)	138.50 [137.00, 141.00]	136.00 [134.00, 139.00]	0.001
Potassium (mEq/L)	4.06 (± 0.53)	4.28 (± 0.43)	0.009
AST (IU/L)	27.00 [22.00, 35.00]	35.50 [24.00, 46.75]	0.004
ALT (IU/L)	18.50 [12.25, 26.00]	23.50 [14.25, 34.00]	0.068
Creatinine (mg/dL)	0.70 [0.60, 0.93]	0.74 [0.60, 0.94]	0.922
Creatinine clearance (mL/min)	63.01 (± 29.26)	62.11 (± 24.96)	0.849
Creatinine clearance categories, n (%)			0.326
Creatinine clearance ≥ 50 mL/min	46 (62.2)	41 (66.1)	
Creatinine clearance 30–49 mL/min	20 (27.0)	16 (25.8)	
Creatinine clearance 15–29 mL/min	3 (4.1)	5 (8.1)	
Creatinine clearance < 15 mL/min	2 (2.7)	0 (0.0)	
Maintenance dialysis	3 (4.1)	0 (0.0)	
eGFR (mL/min/1.73 m^2^)	67.41 (± 27.51)	68.65 (± 22.88)	0.779
Disturbed consciousness, n (%)	1 (1.4)	1 (1.6)	> 0.99
Hypotension (systolic pressure < 90 mmHg), n (%)	0 (0.0)	3 (4.8)	0.092
Oxygen administration			0.406
Without oxygen, n (%)	34 (45.9)	36 (58.1)	
With oxygen, n (%)	38 (51.4)	26 (41.9)	
Mechanical ventilation, n (%)	2 (2.7)	0 (0.0)	
Dose of trimethoprim (mg/kg/day)	12.20 [8.17, 15.72]	14.77 [12.51, 16.95]	0.011
Adjunctive glucocorticoid therapy			
None, n (%)	8 (10.8)	9 (14.5)	0.606
Yes (mild to moderate dose), n (%)	40 (54.1)	30 (48.4)	0.606
Yes (steroid pulse therapy), n (%)	26 (35.1)	23 (37.1)	0.859

Data are expressed as mean (± standard deviation) or median [P27–P75] for continuous variables, and as number (percentage) for categorical variables. P values were calculated using the t-test or Mann–Whitney U test for continuous variables, as appropriate, and Fisher’s exact test for categorical variables. SXT, trimethoprim-sulfamethoxazole; AST, aspartate aminotransferase; ALT, alanine aminotransferase

Table 2 presents the odds ratios obtained using logistic regression analysis of the initial dose of trimethoprim per weight, age, CrCl, absence of steroid treatment, coexistence of malignancy, serum sodium level, and serum potassium level. Notable variations were observed in the initial dose of trimethoprim per weight, serum sodium level, and serum potassium level, with odds ratios of 1.176, 0.884, and 2.646, respectively, and P values of 0.002, 0.020, and 0.023, respectively. No notable variations were observed in age, CrCl, absence of steroid treatment, or absence of malignancy. All VIF values were below 2.0, indicating no serious multicollinearity. The model showed moderate discrimination, with an AUC of 0.76 (95% CI, 0.68–0.84), and good calibration by the Hosmer–Lemeshow test (χ² = 6.36, df = 8, *p* = 0.61). In a sensitivity analysis using the bestglm package with the AIC criterion, serum sodium, serum potassium, and the dose of trimethoprim remained significant predictors, and the model showed similar discrimination (AUC 0.76) and good calibration (Hosmer–Lemeshow *p* = 0.98) (Supplementary Table 4). These findings support the robustness of the main multivariable model presented in Table 2.

**Table 2:** Multivariable logistic regression analysis of risk factors for adverse events associated with trimethoprim-sulfamethoxazole treatment

Variable	Adjusted odds ratio	95% CI	*P* value
Dose of trimethoprim (mg/kg/day)	1.176	1.063–1.309	0.002
Age	0.998	0.950–1.048	0.923
Creatinine clearance	0.995	0.976–1.015	0.626
Non-glucocorticoid therapy	1.146	0.368–3.598	0.813
Underlying disease/malignancy	0.504	0.188–1.279	0.158
Serum sodium level	0.884	0.793–0.978	0.020
Serum potassium level	2.646	1.174–6.418	0.023

Multivariable logistic regression model including the following covariates: dose of trimethoprim, age, creatinine clearance, Non-glucocorticoid therapy, Underlying disease/malignancy, serum sodium, and serum potassium

CI, confidence interval

We compared the occurrence of adverse events between the no treatment failure and treatment failure groups (Table 3). We found notable variations in the incidence of CTCAE grade 3 or higher adverse events, especially skin rashes and nausea, all of which were more common in the treatment failure group. Notable variations were observed in the elevated serum ALT level, which was significantly higher in the no treatment failure group. In the treatment failure group, the most frequent adverse events were skin rashes (27.8%), hyponatremia (26.4%), and hyperkalemia (13.9%). The results were robust in the sensitivity analysis modeling age categorically (< 65 and ≥ 65 years): age group was not independently associated with adverse events (aOR: 0.84 [95% CI: 0.30–2.37]) (Supplementary Table 2).

**Table 3:** Incidence of adverse events in patients with and no treatment failure

Variable	No treatment failure (*n* = 64)	With treatment failure (*n* = 72)	*P* value
Occurrence of grade 3 or higher adverse events, n (%)	16 (25.0)	46 (63.9)	< 0.001
Skin rashes, n (%)	2 (3.1)	20 (27.8)	< 0.001
Nausea, n (%)	0 (0.0)	8 (11.1)	0.007
Leukopenia, n (%)	0 (0.0)	1 (1.4)	> 0.99
Anemia, n (%)	1 (1.6)	1 (1.4)	> 0.99
Thrombocytopenia, n (%)	2 (3.1)	2 (2.8)	> 0.99
Increased serum ALT level, n (%)	5 (7.8)	0 (0.0)	0.021
Hyponatremia, n (%)	8 (12.5)	19 (26.4)	0.053
Hyperkalemia, n (%)	5 (7.8)	10 (13.9)	0.287

ALT, alanine aminotransferase

## Discussion

In this multicenter, retrospective, observational study, we investigated the factors contributing to the occurrence of adverse events in patients with non-HIV PCP treated with SXT. The results indicated that serum sodium level at the beginning of the treatment, serum potassium level at the beginning of the treatment, and initial dosage of SXT were associated with the occurrence of adverse events; a low serum sodium level, a high serum potassium level, and increased initial dosages correlated with an increased risk of adverse events. Additionally, we examined the incidence of adverse events by dividing patients into treatment failure and no treatment failure groups. In the treatment failure group, the incidence of CTCAE grade 3 or higher adverse events was higher, with skin rashes being the most common adverse event.

In the present study, we targeted non-HIV-infected patients with PCP who had various underlying conditions and evaluated the factors associated with adverse events. The results showed that a high initial dose of SXT was associated with an increased risk of adverse events. SXT (trimethoprim, 15–20 mg/kg/day; sulfamethoxazole, 75–100 mg/kg/day) is the first-choice treatment for PCP (according to the guidelines), and its efficacy is well-established [25]. However, adverse events commonly linked to SXT treatment include dermatologic reactions, gastrointestinal symptoms, hematologic suppression, renal and hepatic toxicity, and electrolyte imbalances [6], and half of the patients with HIV PCP have been reported to have had the treatment changed owing to serious adverse events [7, 8]. Chang et al. [6]. demonstrated that high-dose SXT (16 mg/kg/day) administered to patients with HIV PCP was associated with the occurrence of adverse events, consistent with the results of the present study.

In the present study, baseline serum sodium and potassium levels revealed hyponatremia and hyperkalemia associated with SXT administration. Hughes et al. reported that the incidence rates of anemia, neutropenia, and azotemia increased with increasing plasma levels of trimethoprim in patients with HIV PCP. SXT is also known to cause hyperkalemia and hyponatremia [26], and reportedly, the duration of SXT administration and the cumulative dose are associated with increased severity and incidence of hyponatremia [26–29]. In a study involving patients with rheumatoid arthritis and non-HIV PCP, Ohmura et al. [23] reported significantly high electrolyte abnormalities (hyponatremia and hyperkalemia; grade 3 or above) with moderate or high doses of SXT (10 mg/kg/day). However, they did not investigate baseline serum sodium and potassium levels. The present study showed that adverse events were more likely to occur in the presence of hyponatremia and hyperkalemia. SXT also causes hyponatremia and hyperkalemia. It is thought to act as a potassium-retaining diuretic by blocking epithelial sodium channels, causing hyperkalemia and hyponatremia due to natriuresis [30, 31], although the mechanism of these adverse events is not fully understood. Therefore, patients with underlying hyponatremia and hyperkalemia may develop severe electrolyte abnormalities with SXT treatment.

In the present study, the treatment failure group had a higher rate of grade 3 or higher adverse events associated with SXT therapy than the no treatment failure group. We focused on the data concerning adverse events that physicians considered caused by SXT. Therefore, we assumed that these adverse events of SXT were the reasons for discontinuing SXT treatment. Skin rashes, nausea, hyponatremia, and hyperkalemia were the most common adverse events in the treatment failure group. Skin rashes and nausea occurred significantly more frequently in the treatment failure group than in the no treatment failure group. Skin rashes and gastrointestinal symptoms are the most common adverse events associated with SXT treatment [29]. Stevens-Johnson syndrome has been reported as a severe adverse event associated with SXT treatment [32]. Therefore, physicians recognize the occurrence of skin rashes as an adverse event of SXT treatment. Desensitization therapy is a well-established prophylactic method against skin rashes in patients with PCP [33]. However, in therapeutic administration, desensitization therapy cannot be implemented due to the need to raise blood levels of SXT as soon as possible. Therefore, treatment failure is frequent, which might have resulted in a higher frequency of skin rashes in the treatment failure group than in the no treatment failure group. SXT has been reported to cause gastrointestinal adverse events in approximately 3% to 8% of patients, with common symptoms including nausea, vomiting, and anorexia [34]. Nausea of grade 3 or above is considered a burden on the patient and difficult to manage with medication; therefore, SXT treatment is discontinued at the discretion of doctors.

The results of the present study suggest that patients with hyponatremia and hyperkalemia at baseline are at a high risk of developing adverse events. However, the frequency of hyponatremia and hyperkalemia was not significantly higher in the treatment failure group compared to that in the no treatment failure group. Hyponatremia can be treated with salt loading though hyponatremia due to SXT is often difficult to correct by salt loading and may necessitate discontinuation of SXT treatment [22, 29]. Hyperkalemia can often be treated with oral therapy [35] but may require a reduction in the dose of the SXT. Careful management, including regular assessment of serum K level, is important. In addition, low-dose SXT therapy, which has recently been attracting attention [12], is a good option for managing and preventing the abovementioned adverse events.

The present study is the first and largest multicenter study to assess the factors contributing to the adverse effects of SXT treatment in patients with non-HIV PCP. However, this study has several limitations. First, this was a retrospective, observational study. Therefore, the choice of treatment was not random, and confounding by indication bias might have occurred. Second, only a small number of patients, such as those on ventilators, had severe respiratory failure. The factors contributing to the adverse events associated with SXT treatment in patients with severe respiratory failure remain unknown. Third, this study did not include a comprehensive analysis of drug-drug interactions between concomitant medications and SXT. Our cohort comprised patients with serious underlying comorbidities, such as malignancies and autoimmune diseases, who were often receiving multiple medications. Consequently, interactions between these concomitant drugs and SXT may have contributed to the observed adverse events, representing a potential unassessed confounding factor. Fourth, the limitations regarding the precision of data on the causal relationship between adverse events and treatment discontinuation as well as their time course must be acknowledged. The specific causal attribution of adverse events to treatment discontinuation and the precise timing from SXT initiation to the adverse event or discontinuation were not consistently documented across the participating institutions. Furthermore, additional data extraction was not feasible as the dataset was fixed retrospectively, thus precluding a detailed analysis to identify the direct adverse events causing discontinuation and a formal time-to-event analysis. Furthermore, this study evaluated the association between baseline serum sodium/potassium levels and adverse events using these markers as continuous variables; therefore, optimal cutoff values were not determined. Additionally, as we did not systematically collect longitudinal data after SXT initiation, we could not evaluate the temporal patterns of these values according to patient outcomes.

Finally, because the study did not systematically collect data on time-dependent dose modifications or the corresponding therapeutic efficacy and serial laboratory values, we could not definitively identify subgroups that would benefit from an early dose modification strategy aimed at maintaining efficacy while minimizing toxicity. While our results suggest that certain subgroups might benefit from early dose modification, this is purely an extrapolation from our risk factor analysis. Further large-scale, prospective studies are needed to overcome these limitations and establish more robust evidence.

## Conclusions

Pretreatment serum sodium and potassium levels and the initial SXT dose were independent risk factors for developing CTCAE grade 3 or higher adverse events during SXT treatment for non-HIV PCP. Assessment of these risk factors is essential to ensure appropriate SXT treatment and management of adverse events. Further large-scale, prospective studies are needed to validate the present results.

## Supplementary Information

Below is the link to the electronic supplementary material.

Supplementary Material 1

## Data Availability

Data pertaining to this study will be available by the corresponding author upon reasonable request.

## Abbreviations

HIV: Human immunodeficiency virus
PCP: Pneumocystis jirovecii pneumonia
AIDS: Acquired immunodeficiency syndrome
SXT: Trimethoprim-sulfamethoxazole
ALT: Alanine aminotransferase
CrCl: Creatinine clearance
CTCAE: Common Terminology Criteria for Adverse Events
