# Supplementary material for: Risk factors for adverse events associated with trimethoprim-sulfamethoxazole treatment for Pneumocystis pneumonia in non-human immunodeficiency virus-infected patients: a multicenter, retrospective, observational cohort study
Source: BMC Infect Dis. 2026 Jan 20;26:345. doi: 10.1186/s12879-026-12600-7 (PMC12903715; doi:10.1186/s12879-026-12600-7)
Supplement: Supplementary file 1 — Supplementary Material 1 [file 12879_2026_12600_MOESM1_ESM.docx]

**Supplementary Table 1.** Baseline immunosuppressants and biologic immunosuppressive medications and adjunctive corticosteroid use during PCP treatment, stratified by adverse event status

|  | No adverse events  (n = 74) | Adverse events  (n = 62) |
| --- | --- | --- |
| Baseline medications | | |
| Immunosuppressant^a^ | n = 41 | n = 44 |
| Tacrolimus | 1/41 (2.4) | 1/44 (2.3) |
| Cyclosporine | 4/41 (9.8) | 0/44 (0.0) |
| Azathioprine | 1/41 (2.4) | 2/44 (4.5) |
| Cyclophosphamide | 1/41 (2.4) | 0/44 (0.0) |
| Methotrexate | 33/41 (80.5) | 42/44 (95.5) |
| Bucillamine | 5/41(12.2) | 4/44 (9.1) |
| Biologic immunosuppressive drugs | n = 17 | n = 17 |
| Adalimumab | 6/17 (35.3) | 10/17 (58.8) |
| Abatacept | 4/17 (23.5) | 0/17 (0.0) |
| Infliximab | 3/17 (17.6) | 4/17 (23.5) |
| Etanercept | 4/17 (23.5) | 2/17 (11.8) |
| Certolizumab | 0/17 (0.0) | 1/17 (5.9) |
| Use of adjunctive corticosteroids during PCP treatment | | |
| Adjunctive glucocorticoid therapy | n = 66 | n = 51 |
| Methylprednisolone | 11/66 (16.7) | 13/51 (24.5) |
| Prednisolone | 54/66 (81.8) | 40/51 (78.4) |
| Dexamethasone | 1/66 (1.5) | 0/51 (0.0) |

Data are presented as number/total number (%)

^a^Owing to patients receiving multiple agents, column totals may exceed the number of patients.

PCP, *Pneumocystis jirovecii* pneumonia

**Supplementary Table 2.** Unadjusted odds ratios obtained using logistic regression analysis

| Variable | Unadjusted odds ratio | 95% CI | P value |
| --- | --- | --- | --- |
| Age | 0.992 | 0.961 – 1.020 | 0.635 |
| Age ≥65 years | 0.914 | 0.425 – 1.970 | 0.818 |
| Dose of trimethoprim (mg/kg/day) | 1.180 | 1.080 – 1.300 | 0.0004 |
| Non-glucocorticoid therapy | 1.400 | 0.502 – 3.970 | 0.517 |
| Underlying disease | | | |
| malignancy | 0.519 | 0.215 – 1.190 | 0.130 |
| connective tissue disease | 1.550 | 0.721 – 3.410 | 0.269 |
| Creatinine clearance | 0.999 | 0.986 – 1.010 | 0.847 |
| Baseline medications | | | |
| Glucocorticoid | 0.702 | 0.350 – 1.400 | 0.317 |
| Immunosuppressant | 1.970 | 0.971 – 4.080 | 0.064 |
| Biologic immunosuppressive drugs | 1.270 | 0.580 – 2.770 | 0.551 |
| Antineoplastic agents | 0.919 | 0.310 – 2.630 | 0.875 |
| Serum sodium level | 0.860 | 0.779 – 0.939 | 0.001 |
| Serum potassium level | 2.590 | 1.270 – 5.610 | 0.012 |

CI, confidence interval

| Variable | Odds ratio | 95% CI | P value |
| --- | --- | --- | --- |
| Dose of trimethoprim (mg/kg/day) | 1.170 | 1.06 - 1.31 | 0.002 |
| Age ≥65 years ^a^ | 0.840 | 0.30 - 2.37 | 0.740 |
| Creatinine clearance | 0.994 | 0.98 - 1.01 | 0.515 |
| Non-glucocorticoid therapy | 1.170 | 0.37 - 3.68 | 0.789 |
| Underlying disease/malignancy | 0.508 | 0.19 - 1.28 | 0.160 |
| Serum sodium level | 0.882 | 0.79 - 0.98 | 0.018 |
| Serum potassium level | 2.630 | 1.17 - 6.39 | 0.024 |

**Supplementary Table 3.** Adjusted odds ratios from multivariable logistic regression including age category (<65 and ≥65 years)

CI, confidence interval.

^a^ The reference category for age is <65 years.

**Supplementary Table 4.** Adjusted odds ratios from multivariable logistic regression models selected using the bestglm package

| Variable | Odds ratio | 95% CI | P value |
| --- | --- | --- | --- |
| Dose of trimethoprim (mg/kg/day) | 1.180 | 1.06 - 1.31 | 0.002 |
| Use of antineoplastic agents | 3.55 | 0.632 - 23.7 | 0.163 |
| Underlying disease/malignancy | 0.238 | 0.047 – 0.928 | 0.053 |
| Serum sodium level | 0.880 | 0.799 - 0.982 | 0.024 |
| Serum potassium level | 2.73 | 1.21 - 6.76 | 0.020 |

CI, confidence interval.
